# Intracellular detection and communication of a wireless chip in cell

**DOI:** 10.1038/s41598-021-85268-5

**Published:** 2021-03-16

**Authors:** Mimi X. Yang, Xiaolin Hu, Demir Akin, Ada Poon, H.-S Philip Wong

**Affiliations:** 1 Department of Electrical Engineering, Stanford University, Stanford, CA 94305 USA; 2 Center for Cancer Nanotechnology Excellence, Stanford University, Stanford, CA 94305 USA; 3 Chan Zuckerberg Biohub, San Francisco, CA 94158 USA

**Keywords:** Biomedical engineering, Electrical and electronic engineering

## Abstract

The rapid growth and development of technology has had significant implications for healthcare, personalized medicine, and our understanding of biology. In this work, we leverage the miniaturization of electronics to realize the first demonstration of wireless detection and communication of an electronic device inside a cell. This is a significant forward step towards a vision of non-invasive, intracellular wireless platforms for single-cell analyses. We demonstrate that a 25 $\upmu$m wireless radio frequency identification (RFID) device can not only be taken up by a mammalian cell but can also be detected and specifically identified externally while located intracellularly. The S-parameters and power delivery efficiency of the electronic communication system is quantified before and after immersion in a biological environment; the results show distinct electrical responses for different RFID tags, allowing for classification of cells by examining the electrical output noninvasively. This versatile platform can be adapted for realization of a broad modality of sensors and actuators. This work precedes and facilitates the development of long-term intracellular real-time measurement systems for personalized medicine and furthering our understanding of intrinsic biological behaviors. It helps provide an advanced technique to better assess the long-term evolution of cellular physiology as a result of drug and disease stimuli in a way that is not feasible using current methods.

## Introduction

**Figure 1:**
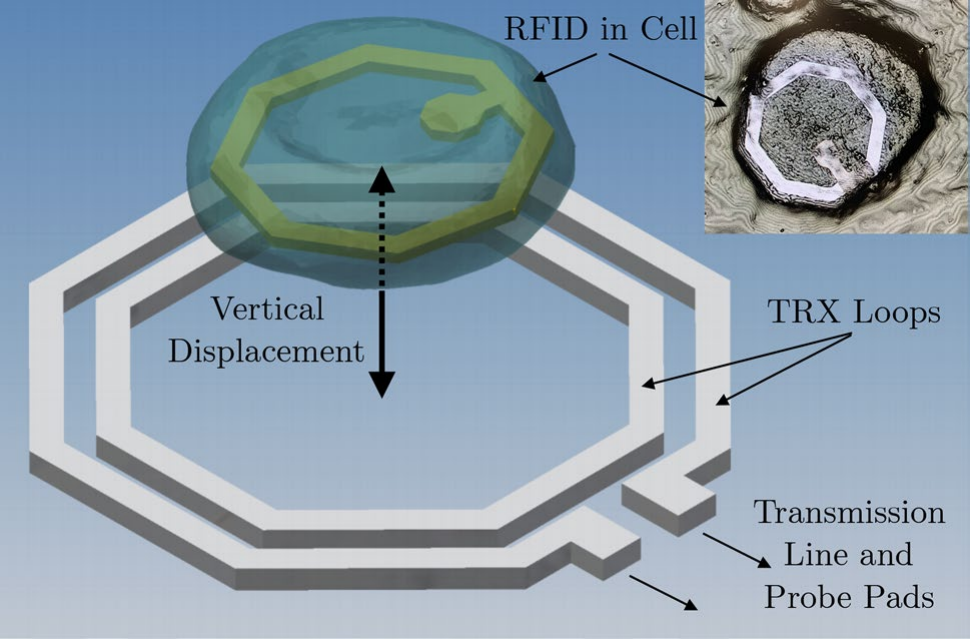
Cell containing intracellular RFID aligned within transceiver antenna loops for RFID detection and identification. The transceiver antennas are connected to a transmission line and probe pads used for electrical measurements. The insert shows an optical image of the RFID in a cell taken with a Keyence VK-X Series 3D Laser Scanning Confocal Microscope.

Today’s healthcare is primarily focused on analyzing and treating patients after the manifestation of disease^1^. The rapid growth and development of technology enables the capability to actively monitor well-being of the individual and focus on prevention rather than episodic reaction. This field of precision medicine is augmented by the ability to develop sensing technologies for continuous monitoring of physiological changes and biochemistry, and the ability to communicate and interpret that information in a timely manner. There are many levels of granularity at which this sensing and monitoring can occur, for example, body level^1–3^, organ level^4,5^, and tissue level^6,7^. The wireless platform reported here offers a new, electronics-based capability to do so at the cellular level. This work enables future ability to measure biomarkers and general physiology within cells and potentially interactions between cells.

The vision for technologies operating at the cellular level is to non-invasively sense and actuate within cells, primarily for biomedical applications and furthering our understanding of biology^8,9^. In recent years, there have been a multitude of attempts^10^ to conduct intracellular measurements with passive lab-synthesized nanoparticles^11–14^, photonic sensors^15^, fluorescent tags^16–18^, and wired microstructure probe arrays^19–25^. While there have been various adaptations of these platforms for different applications, each technology requires significant effort to modify it for the target applications. In contrast, an electronic based system benefits from the microfabrication capabilities developed over time to quickly design and integrate a custom solution. Additionally, the existing platforms are often only operable in very specific experimental settings. Common limitations include incompatibility with optically opaque environments, inability to do in vivo testing, and very slow measurement throughput.

Our goal in this work is to create a versatile intracellular system that can easily be customized to measure a variety of parameters in real time such as intracellular pressure, pH, ion levels, and biomolecule presence. Such a universal system could offer long-term measurements and may eventually be implanted in vivo. We take advantage of microfabrication, wireless communication, and power transfer abilities of radio frequency (RF) technology to develop a system that has the potential to be a powerful tool in developing personalized medicine designed for maximum efficacy and characterizing and predicting biological behaviors under different stimuli like drugs and disease. We strive to develop long-term monitoring capabilities to capture some of these cellular physiological changes that may occur over time. This system architecture uniquely permits the parallel development of a wide modality of sensor interfaces (chemical, biological, electrical, mechanical, etc.) to be integrated with the universal platform.

## Results

Our RF systems includes two main components, an implantable radio frequency identification (RFID) device and an extracellular transceiver (TRX) detection component. The role of the transceiver is to excite, detect, and uniquely identify the RFID tag implanted in a cell. The minimum size of the RFID tag is constrained by the RF antenna, which is typically with dimensions on the order of a quarter wavelength of the operating frequency for far-field detection. For this application, far-field communication requires operation at optical frequencies, which limits future in vivo operation. Thus, a subwavelength RFID design operating at near field is required for the RFID to be implantable inside of cells and for millimeter wavelength RF communication. So far, the most similar and smallest realization of implanted electronic chips has been 400 $\upmu$m RFID chips (commercially produced) embedded in organelles^26^.

Figure 1 shows a rendering in which individual cells are tagged with the RFID tags. We^27^ modeled, designed, and fabricated an aseptic version of this miniature wireless system using Taiwan Semiconductor Manufacturing Company (TSMC) 40 nm complementary metal–oxide–semiconductor (CMOS) technology^28^. The dimensions and design constraints are contingent on the size-dependent internalization rates of the RFIDs by cell, as previously revealed by Chen^29^. It has also been demonstrated that the RFID tags fabricated for the intracellular system are taken up at greater rates by various cancer cell lines relative to healthy cells, with 60–70% of tags typically uptaken within 24–48 h by melanoma cells used in this study^30^.

**Figure 2:**
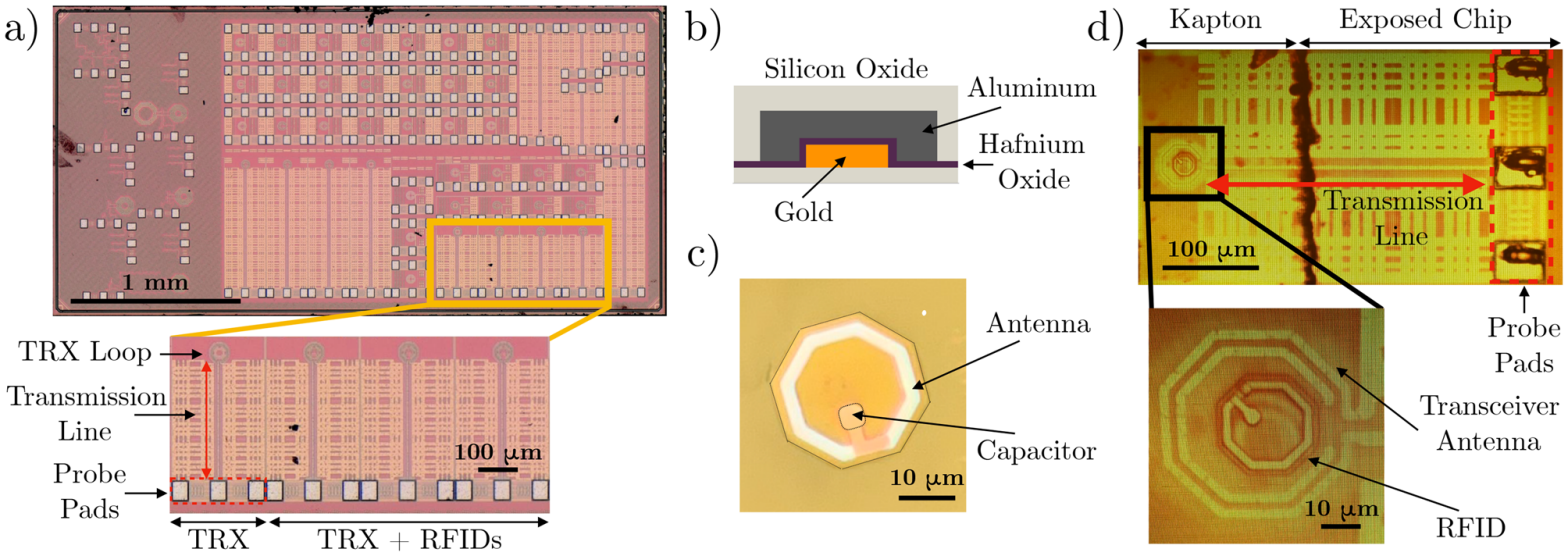
Hardware components of the intracellular detection system. (**a**) Transceiver structures are fabricated on an integrated circuit. Each chip includes a standalone transceiver and three transceivers with concentric, integrated RFIDs. Each transceiver loop is connected to a 300 $\upmu$m transmission line and probe pads. (**b**) Schematic cross section of RFIDs fabricated in the Stanford Nanofabrication Facility (SNF). (**c**) Top view of a loose RFID device. The device consists of an antenna (white outer ring) and a capacitor (square feature situated inside the ring), whose size and value can be modified during fabrication to generate different RFID batches with different electrical characteristics. (**d**) Using Kapton film as a carrier, individual RFIDs can be placed within the footprint of the transceiver antenna to be detected and identified. The probe pads must be uncovered to perform electrical measurements.

The electrical characteristics of the subwavelength RFID design were first quantified at the chip level^27^, with RFIDs and transceiver fabricated together in a single configuration. Specifically, there are four structures of interest within the integrated circuit, as shown in Fig. 2a. They include a standalone transceiver structure and three transceiver structures with different RFID designs integrated concentrically within the sensitive detection zone of the transceiver. The three transceiver structures each contains a RFID structure (Fig. 2c) fabricated on the same silicon substrate, and each RFID possesses a different capacitor size and electrical characteristics. Each transceiver structure consists of two transceiver loops connected to a 300 $\upmu$m transmission line, which is connected to probe pads. The characteristics of the electronic devices are represented by their S-parameter reflection coefficient ($S_{11}$). The $S_{11}$ quantifies the efficiency of power delivery to the antenna, with resonant frequencies indicating the most efficient operating points. Note that with the introduction of cell bodies and discrete RFID devices fabricated in a separate, in-house process (Fig. 2b and c), there is variability introduced to the RFID placement and electrical performance compared to the measurements conducted at the chip level.

Arrays of individual RFIDs are fabricated on silicon wafers in the Stanford Nanofabrication Facility (SNF). This allows the mass fabrication of passive metal-oxide-metal structures RFID devices. The device structure consists of an aluminum antenna loop and a capacitor with aluminum and gold plates and hafnium oxide dielectric. The entire structure is encapsulated in silicon dioxide for biocompatibility (Fig. 2b). The individual RFIDs are released from the wafer carrier using a xenon difluoride etching process. For the full fabrication process, see the works by Hu and Chen^29,31^. The updated process used in this work involves washing the wafers with isopropyl alcohol rather than phosphate-buffered saline (PBS) to create a mixture solution with RFIDs.

After fabricating these loose, 25 $\upmu$m devices, we require a precise and repeatable alignment process to verify the baseline electrical performances of the RFID devices. Hu et al. simulated the sensitivity of the RFID detection to the range of its placement with respect to the transceiver loop^27^. It was determined that the highest electrical sensitivity is achieved when the RFID is placed fully within the transceiver loop. Thus, we are motivated to replicate this configuration experimentally to realize the largest possible electrical measurement change. Due to small sizes of the RFIDs and transceiver loops, manual placement and alignment is difficult. Rather than handling individual devices, we dispense a droplet of the RFIDs in isopropyl alcohol onto a 5 mm × 5 mm sheet of 7.6 $\upmu$m thick Kapton Film (CS Hyde) coated with a thin layer of semi-cured polydimethylsiloxane (PDMS). After the isopropyl alcohol evaporates, we end up with a carrier film of Kapton coated with a random dispersion of RFIDs. Concurrently, the integrated chip is embedded in a sheet of PDMS to aid handling. Using a flip chip bonder, the entire Kapton film can be picked and placed relative to the transceiver feature on the integrated chip with sub-micron accuracy (Fig. 2d). With the development of this alignment and placement technique, it is now possible to have an accurate and repeatable setup to conduct high sensitivity electrical measurements on individual, loose RFIDs.

Prior to implanting the RFIDs inside the cells, it is important to understand how the interactions between the electronics and biology will affect the measured system characteristics. Primarily, electronics are fabricated and operated in sterile environments. With the introduction of biological cells and dynamic, organic environments, there likely are unexpected changes in the electrical characteristics measured to detect and identify the RFID tags^32^. The performance changes can be estimated and characterized with a proxy of soaking the RFIDs in a PBS environment to quantify the amplitude and frequency shifts of the characteristic resonance response. The degradation patterns of the electrical signal due to the biological fluid is illustrated in Supplementary Fig. S1. The results confirm that the resonance features of the electrical signal, which are used to identify and distinguish between different RFIDs, remain distinct after 24 h in the PBS solution. This is the amount of time needed for the cell uptake process of the RFIDs to reach equilibrium, during which there are no biocompatibility issues associated with the fabricated RFID tags and uptake procedure^29^. The source of the electronic degradation is the diffusion of ions through the silicon oxide encapsulation, which can change the device capacitance. This capacitance is directly related to the values of the resonance frequencies. It is expected that minimal signal degradation is attributed to changes in the inductance of the antenna loop. We observe no visible corrosion or deterioration in the antenna material that would suggest a change in RFID inductance characteristics during the duration of this study. To prevent such device degradation before the cell uptake experiments, the RFIDs are washed and stored in isopropyl alcohol rather than PBS, as described in previous works^29,30^.

**Figure 3:**
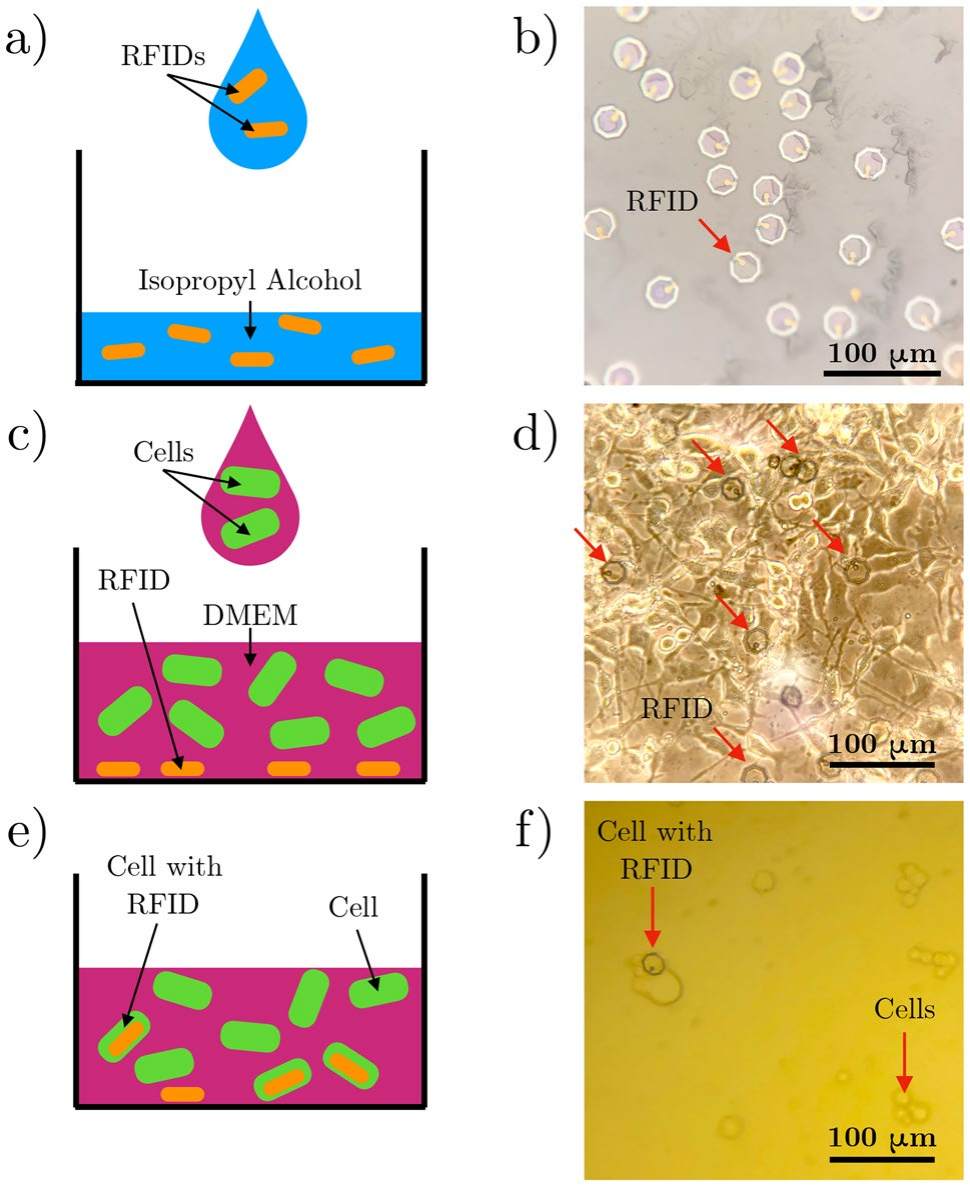
RFID internalization process procedure. (**a**) Drop an aliquot of the RFID solution into a cell well. (**b**) Once the isopropyl alcohol evaporates, the RFIDs remain at the well bottom. (**c**) Dispense a suspension of cells on top of the RFIDs. (**d**) After 24 h, many RFIDs are internalized inside the cells. In this microscope image of cultured cells, the arrows point to the RFIDs. (**e**) Dissociate the cells and RFID culture with Trypsin, as described in the methods section. (**f**) The result is a suspension of cells and cells with implanted RFIDs.

With the validation of the electrical system in a synthetic test environment and the understanding of the degradation patterns of the devices in a biological environment, the final intracellular measurements can be performed. The storage of the devices in isopropyl alcohol requires modification of the previously established cell-uptake-procedure to prevent cell death due to alcohol toxicity. To do so, the RFID solution is dispensed into a cell culture well and the isopropyl alcohol carrier liquid is allowed to evaporate (Fig. 3a and b). A cell suspension harvested from an adjacent, 80% confluent well is deposited on top of the RFIDs and allowed to attach and uptake the RFIDs over 24 h (Fig. 3c and d). The RFID and cells can be harvested to create a suspension of cells, some of which have engulfed RFIDs, as shown in Fig. 3e and f.

**Figure 4:**
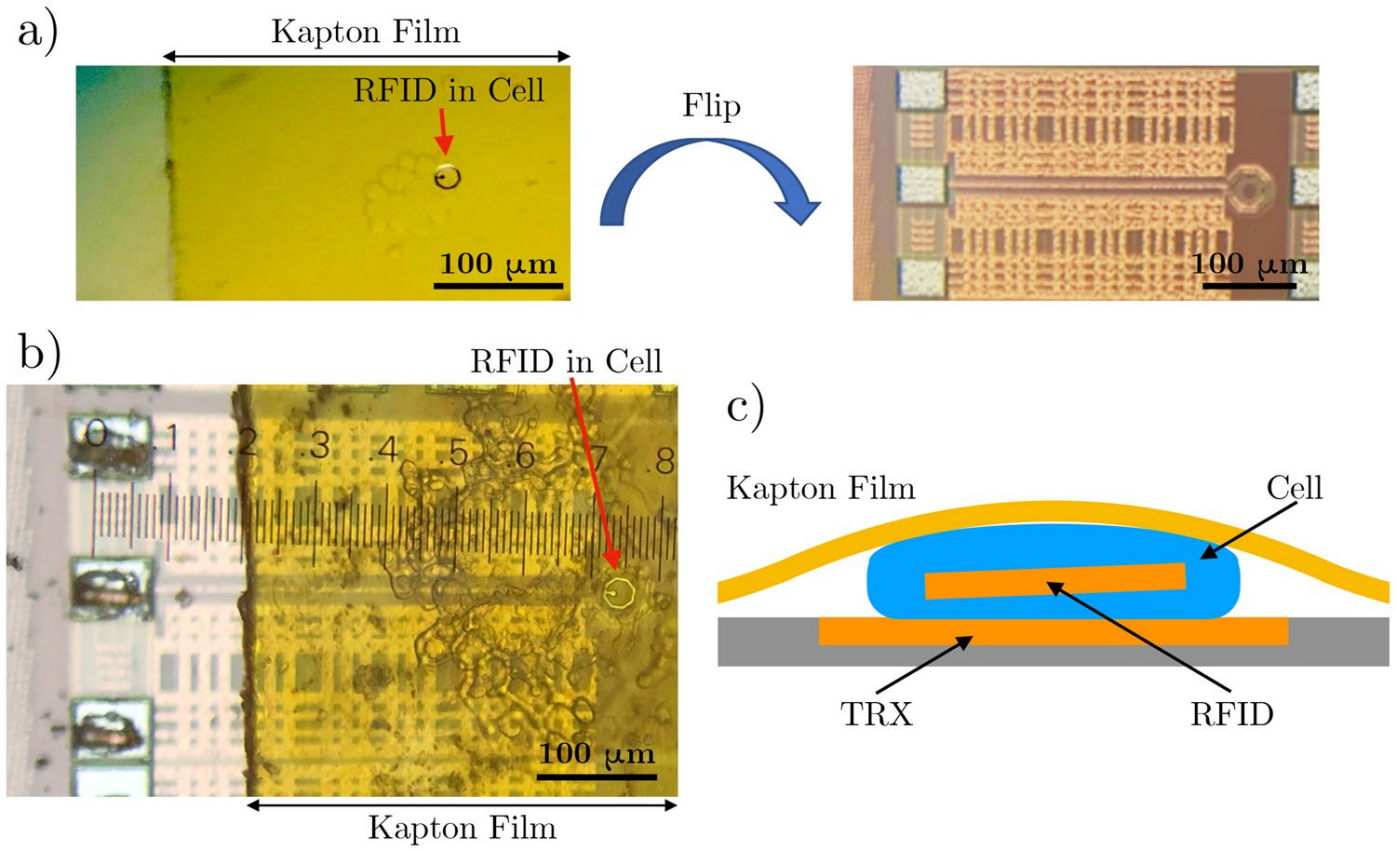
Cell and RFID alignment onto a transceiver. (**a**) Using the flip chip bonder, flip a Kapton film with a RFID in a cell onto the transceiver structure on the integrated circuit chip. (**b**) RFID is aligned in the transceiver loop, and probe pads are uncovered for electrical measurements. (**c**) Cross section schematic shows that the Kapton film pins the cell and intracellular RFID within the transceiver detection zone.

The cell suspension can be treated in a similar manner as the original RFID solution for testing purposes. Specifically, an aliquot of the cell suspension can be dispensed onto a Kapton film, which can then be placed and aligned onto the transceiver feature (Fig. 4a). In Fig. 4a, we see an intracellular RFID in a clump of cells. That sample is flipped and placed onto the transceiver chip, resulting in the configuration shown in Fig. 4b. This allows for a stack that consists of a cell with RFID sandwiched between the transceiver loop feature and the Kapton film, as shown in Fig. 4c. The Kapton film secures the cell to the surface of the chip similar to a coverslip. The electrical measurements are conducted immediately after the alignment process.

**Figure 5:**
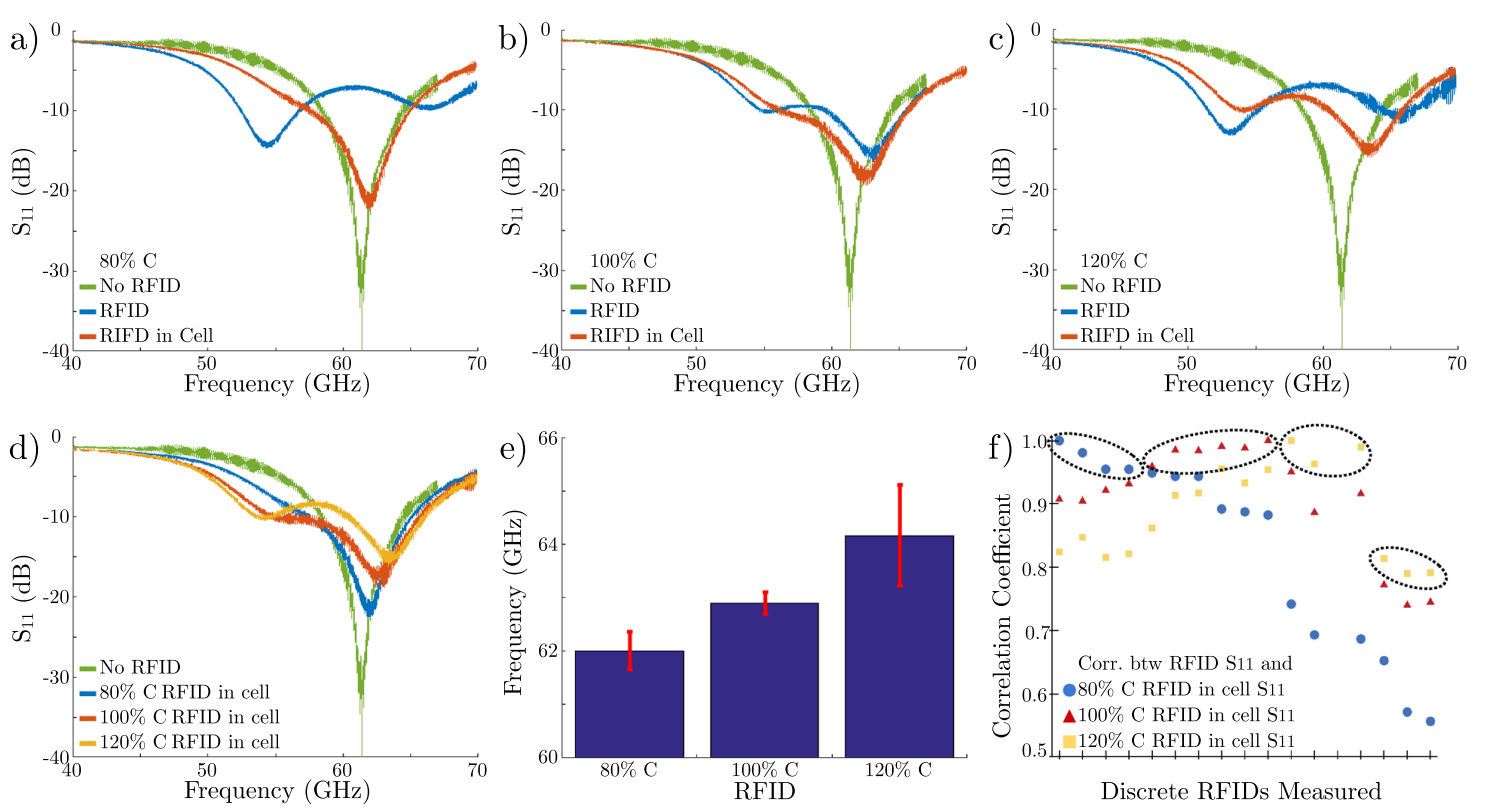
(**a**–**c**) Electrical response of the standalone transceiver (green) and the three different classes of RFIDs (80%C, 100%C, and 120%C) stored in isopropyl alcohol (blue) and 24 h after completing the uptake process (red). (**d**) A direct comparison of the electrical responses of the three batches of intracellular RFIDs 24 h after completing the uptake process. The different devices show different electrical signatures with different resonant frequencies. (**e**) Mean and standard deviation of the resonant frequency of a sample of 5 devices for each capacitor value RFID. (**f**) Given a mixed batch of RFIDs with all three types of devices, individual RFIDs can be categorized according to the correlation match between the $S_{11}$ response and the expected RFID $S_{11}$ responses. The x-axis designates individual RFID measurements and the y-axis represents the correlation coefficient calculated from the measurements. Note that high correlation (circled) indicates the measured RFID’s most probable capacitance and corresponding design, enabling us to identify individual RFID tags.

Three batches of RFIDs were fabricated. These devices differed in the integrated load capacitance (80%C, 100%C, 120%C where nominal C is 0.27 pF^27^), which produces different $S_{11}$ frequency profiles. Specifically, the capacitors of the RFIDs, as seen in Fig. 2c, have three possible sizes and corresponding capacitance values. The RFID designs fabricated in the SNF are identical to those that were integrated with the transceivers on the integrated chip (Fig. 2a), which were previously measured by Hu^27^ and set the $S_{11}$ baseline measurement. Prior to the cellular uptake, the different batches of devices had distinct $S_{11}$ profiles, characterized by the values of the resonance frequencies. After the cell uptake process, it is crucial to quantify whether the RFIDs could still be detected and whether the different batches of devices could be separated and identified. Figure 5a–c depicts the baseline $S_{11}$ profiles of the loose RFIDs and the respective profiles after 24 h in cell cultures. It is clear that all three batches of devices experienced a change in the electrical signature due to the electronic-biologic interactions as predicted in the device degradation tests. We observe a shift in resonance frequencies and some changes in amplitude. One batch of devices experienced a greater relative change of $S_{11}$. This can be explained by the fact that the batch has the lowest, absolute capacitance by design (80%C). Thus, the highest relative change in capacitance is due to the diffusion of ions from the cell culture solution, which correlates to the largest $S_{11}$ change.

These results demonstrate the capability to detect and communicate with intracellular RFIDs, and it is important to be able to identify and differentiate them. Namely, are the $S_{11}$ profiles distinct after the frequency and amplitude changes due to the ion diffusion? Figure 5d shows a stacked graph of the three intracellular measurements. All three curves exhibit a similar two peak curvature due to mode splitting, with a predominant resonant peak. These main resonance peaks are distinctly separate, demonstrating that the resonance frequencies are identifiable. As the device capacitance increases, the main resonance frequency increases with decreasing magnitude, in a trend similar to that presented in the reference RFIDs that are not exposed to biological environments.

To make this technology useful in real world applications, it is important to demonstrate repeatability and characterize the spread of electrical performance. Since we have demonstrated that the main resonance peak frequency can be used to identify the originating batch, we sampled five intracellular devices from each design to gather statistics. Figure 5e shows the mean and standard deviation of the resonance frequency. The average resonance frequencies are 61.8, 63.1, 65.5 GHz for the 80%C, 100%C, and 120%C devices, respectively. Therefore, the frequencies are well separated by approximately 1.3 GHz. The distribution of frequencies show low variability for the 80%C and 100%C devices. The larger variability of the 120%C design may be due to variation in the fabrication process. Since all the discrete RFIDs were fabricated at the Stanford Nanofabrication Facility, which is not a production level facility, we expect cross-wafer variations and wafer-to-wafer variations. In addition, the dynamic ionic concentrations in the cell cultures may introduce additional variations in electrical characteristics, depending on the cell density in the culture plate.

To increase our confidence in the ability to distinguish between the three sets of RFID designs, we use autocorrelation to assess the correlation between the measurements and the RFID designs. Autocorrelation is used to determine the degree of similarity between two signals. Calculating this coefficient between the $S_{11}$ of a RFID of unknown capacitance and the reference $S_{11}$ of the 80%C, 100%C, and 120%C RFID devices reveals the most probable capacitance and design of the RFID, with a larger coefficient implying a better match. Given a solution of all three device designs mixed together, Fig. 5f illustrates the use of the linear correlation coefficient to determine the degree of match with the profiles shown in Fig. 5d. Each measurement clearly shows a higher match to a single $S_{11}$. There are clear profile matches (>90%C) for the 80%C and 100%C devices, while some of the 120%C devices exhibit lower match percentages. This can be attributed to the larger variations in the resonance frequency previously shown in Fig. 5e. More sophisticated signal processing techniques can be employed to match electrical responses measured in dynamic applications with their corresponding design parameters. This enables correlation of changes in the $S_{11}$ from vertical and lateral RFID displacements (Supplementary Fig. S2).

## Discussion and conclusion

In this work, we have demonstrated a chip in cell wireless platform that sets the foundation of future, high impact intracellular detection systems. This wireless platform is universal, allowing for sensor and actuator integration to develop tools for personalized medicine and understanding intrinsic biological behavior. A wide range of interfaces that converts physiological changes to deltas in capacitance or charge changes can be integrated to resolve biological questions. Future work includes improving the device encapsulation with silicon carbide to prevent degradation^33^ and incorporating the electronics into a microfluidic system for a high throughput, intracellular detection system^34^.

With a more robust electronics platform, effort can be devoted to the integration of this system with sensor and actuator interfaces. The integration of CMOS compatible solid-state sensors and actuators can enable measurement of biological parameters such as intracellular pressure, action potentials, and ion concentrations. Engineering pH sensitive hydrogels to regulate the capacitor of the RFID is another pathway to monitor intracellular pH. Incorporating these sensing modalities will enhance the functionality of the toolbox used for cancer cell studies. Also, it is of great interest and importance to explore the possibility of embedding microfabricated structures to actuate/destruct cells, especially cancer cells. This is likely accomplished with the use of high-voltage CMOS circuits that may be able to self-destruct the RFID and the cancerous cell upon detection. There is significant work required prior to achieving the full potential of this intracellular RFID platform.

## Methods

### RFID release

The RFID array was fabricated on the wafer substrate with a release ring etched through the silicon oxide and hafnium oxide encapsulation. A xenon difluoride ($XeF_2$) etch was then used to separate the devices from the carrier wafer. The $XeF_2$ gas etches the silicon wafer material under the individual devices, releasing each RFID into a well on the surface of the carrier wafer. The RFIDs can then be collected into solution by washing the carrier wafer with isopropyl alcohol, and then the solution was collected in a centrifuge tube. After the high-density devices settled to the bottom of the tube, the RFID concentration was increased with the removal of excess isopropyl alcohol. Using gravity rather than a centrifuge to separate the RFIDs to the bottom of the tube and to increase the device concentration limits the potential stress and damage inflicted on the electronics. The remaining solution was then pipetted into a glass chromatography vial for stable stock solution storage.

### RFID internalization

To prepare for the RFIDs to be internalized by cells, an aliquot of the RFID solution was pipetted into a single well of a 24-well cell plate. The isopropyl alcohol was allowed to evaporate, leaving only the electronic solids in the well. In parallel, mouse melanoma cells were grown to 80% confluence in a separate well of the cell culture plate. After washing the cells with a 1% w/v bovine serum albumin (BSA) in phosphate-buffered saline (PBS) solution, the cells in the well were dissociated with a 5-min incubation of 200 $\upmu$L of Trypsin-EDTA (0.05%). To quell the reaction, 1 *mL* of 10% fetal bovine serum (FBS) supplemented with Dulbecco’s Minimal Essential Media (DMEM) was added to the cells. With the cells dissociated, the cell suspension was pipetted into the well containing the RFIDs (with a RFID-to-cell ratio of 1:40) and incubated for 24 h at 37$^{\circ }$C with 5% CO$_2$.

### Creating sheets of cell and RFID samples

A cell and RFID suspension was created by dissociating the cultured cells that were internalizing RFIDs for 24 h from the cell well. A Trypsin-EDTA (0.05%) and 10% FBS supplemented DMEM solution was used to create a suspension of cells. To prepare the sample substrate, 5 mm × 5 mm sheets of 0.3 mil thick Kapton Film (CS Hyde) were cut and plasma treated with 10 sccm of oxygen gas at 35 Watts for 30 s. 100 $\upmu$L of the cell suspension was dropped on the treated Kapton surface and excess liquid was allowed to evaporate.

### Embed chip die

Please refer to Supplementary Fig. S3. The transceiver chip was first thinned to a thickness of 150 $\upmu$m (Aptek Industries, Inc.). To mount the chip, a 2 inch × 2 inch sheet of double-coated REVALPHA thermal release tape (Nitto) was applied with the pressure-sensitive side adhered to a silicon carrier wafer. The liner for the thermal-release side was then removed and the transceiver chip was firmly placed, feature side down, into the middle of the tape segment. A 10:1 (weight:weight) base:curing agent mixture of polydimethylsiloxane (PDMS) was then poured onto the chip and tape and spin coated at 400 rpm for 30 s. The entire sample was then cured at 70$^\circ C$ for 20 min. After curing, a razor blade was used to cut the PDMS just within the edges of the tape, and excess PDMS on the wafer was removed. To release the chip, the wafer was heated on a hotplate at 130$^\circ C$ until the tape changed color and the embedded chip lifted easily from the tape. To provide a more rigid backing, the chip and PDMS film was adhered to a glass slide, electronic feature side up.

### Sample alignment and placement

A 2 mm × 2 mm vacuum head was selected for a FINEPLACER lambda flip chip bonder. The head vacuum piece can then be used to pick up the 5 mm × 5 mm piece of Kapton with cells. The embedded transceiver chip in PDMS was then placed on the base vacuum plate to prepare for alignment. The Kapton and chip were aligned so that the RFID in cell was placed in the transceiver loop center and the probe pads remained uncovered. The two surfaces were contacted with zero force, and releasing the head vacuum resulted in a precisely placed RFID sample on the electronic die substrate.

## Supplementary Information

Supplementary Figures.
