# Supplementary material for: Intracellular detection and communication of a wireless chip in cell
Source: Sci Rep. 2021 Mar 16;11:5967. doi: 10.1038/s41598-021-85268-5 (PMC7966781; doi:10.1038/s41598-021-85268-5)
Supplement: Supplementary file 1 — Supplementary Figures. [file 41598_2021_85268_MOESM1_ESM.pdf]

Supplemental Materials: Intracellular Detection and Communication of a Wireless Chip in Cell

Mimi X. Yang, Xiaolin Hu, Demir Akin, Ada Poon & H.-S. Philip Wong

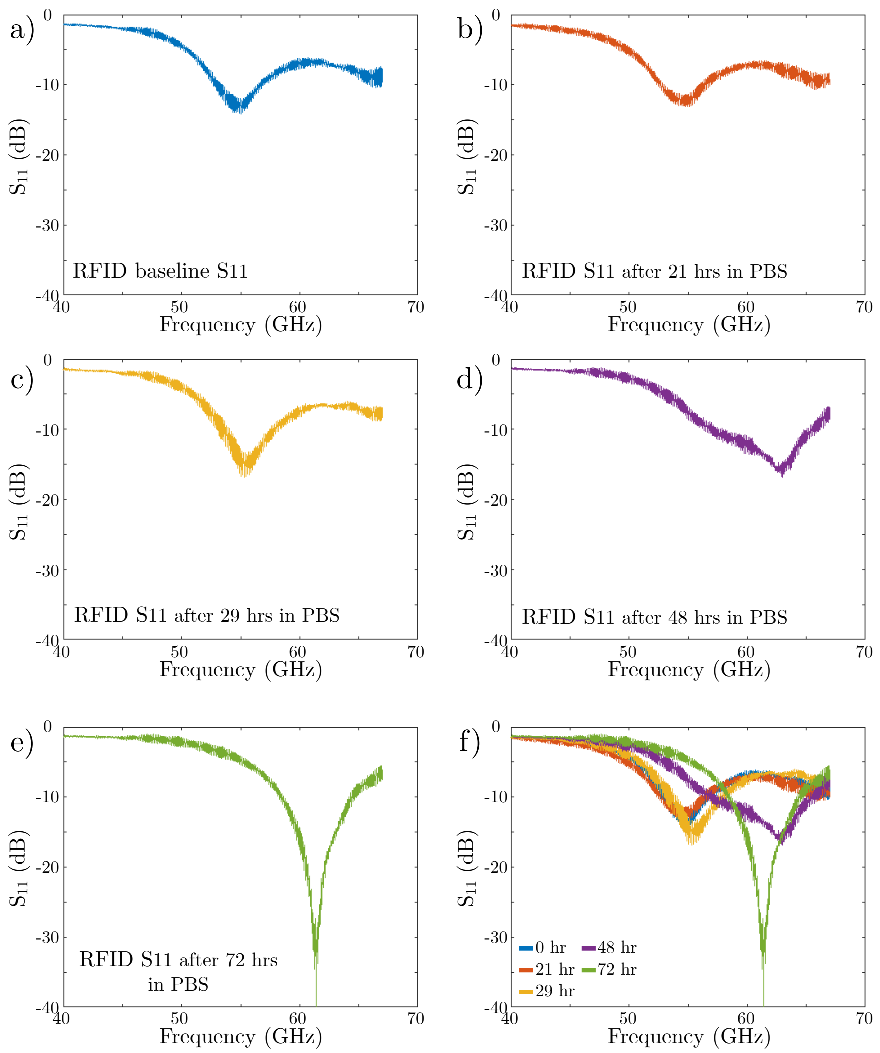

Supplementary Figure S1: Degradation of discrete RFID's measured S_{11} signal as a function of immersion time in PBS solution. a)-e) show the electrical characteristics of loose RFIDs after soaking in PBS for 0, 21, 29, 48, and 72 hours respectively. f) Stacking the measurements detailed in a)-e) highlights the changes in resonance frequency due to diffusion of PBS ions into the device structure. The ion diffusion alters the material characteristics of the device, which causes changes in the electrical characteristics. At 72 hours, the S_{11} profile is identical to that of the transceiver loop alone. Thus, the current device design is best utilized within 48 hours, which allows for detection and identification of RFIDs based on the obvious resonance features.

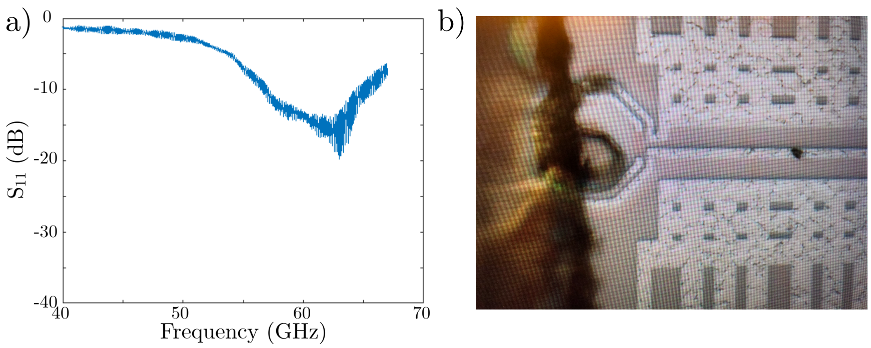

Supplementary Figure S2: Detection of a discrete RFID that has vertical and lateral displacement relative to the transceiver detection loop. a) The S_{11} characteristics see a shift in the resonance frequency, as simulated and predicted by Hu et al. [27]. b) Optical image shows a placed RFID that is at an angle and off center relative to the transceiver loop.

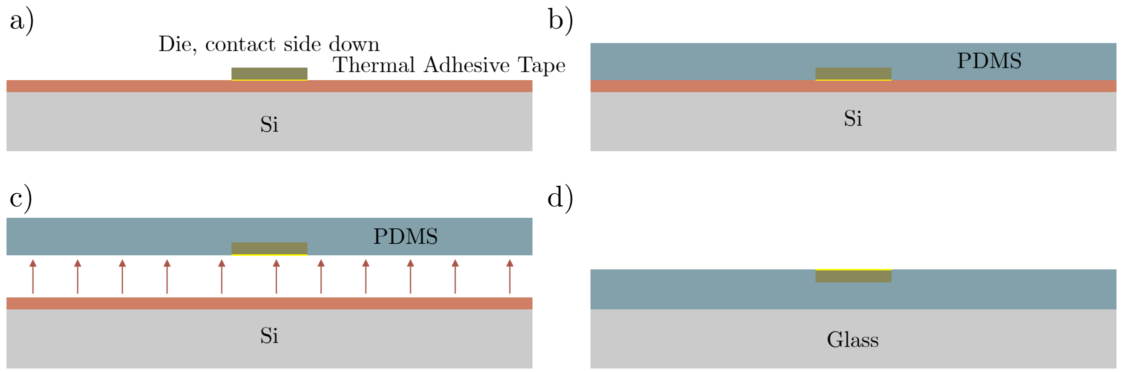

Supplementary Figure S3: Silicon chip embedding process. This process embeds a chip into a layer of PDMS so that the device surface is exposed and extended. This result has the native chip surface flush with the surrounding PDMS. This enables easier chip handling. a) Attach a double sided thermal release tape to a silicon wafer. Press the device side of a thinned chip onto the thermal release side of the tape. b) Pour and cure 200 μm of PDMS on the chip and tape surface. c) Heat the wafer up to 120°C to release the embedded chip. d) Flip the PDMS layer and attach to a supporting glass wafer.
